# Cardiovascular Health and Related Health Care Use of Moluccan-Dutch Immigrants

**DOI:** 10.1371/journal.pone.0138644

**Published:** 2015-09-22

**Authors:** Tim R. de Back, Adee J. Bodewes, Lizzy M. Brewster, Anton E. Kunst

**Affiliations:** 1 Department of Public Health, Academic Medical Center—University of Amsterdam, Amsterdam, the Netherlands; 2 Department of Internal and Vascular Medicine, Academic Medical Center—University of Amsterdam, Amsterdam, the Netherlands; Morehouse School of Medicine, UNITED STATES

## Abstract

**Objective:**

Studies regularly show a higher incidence, prevalence and mortality of cardiovascular disease among immigrant groups from low-income countries. Despite residing in the Netherlands for over 60 years, the Moluccan-Dutch cardiovascular disease profile and health care use are still unknown. We aimed to compare (a) the clinical prevalence of cardiovascular diseases and (b) the use of health care services by cardiovascular disease patients of 5,532 Moluccan-Dutch to an age-sex matched control group of 55,320 native Dutch.

**Methods:**

We performed a cross-sectional analysis of data of the Achmea health insurance company for the period of 1 January 2009 to 31 December 2010. We collected information on health care use, including diagnostic information. Linear and logistic regression models were used for comparison.

**Results:**

Moluccans had a higher clinical prevalence of ischemic heart diseases (odds ratio 1.26; 95% confidence interval 1.03–1.56), but tended to have a lower prevalence of cerebrovascular accidents (0.79; 0.56–1.11) and cardiac failure (0.67; 0.44–1.03). The clinical prevalence of cardiovascular diseases together tended to be lower among Moluccans (0.90; 0.80–1.00). Consultation of medical specialists did not differ. Angiotensin II inhibitors (1.42; 1.09–1.84), antiplatelet agents (1.27; 1.01–1.59) and statins (1.27; 1.00–1.60) were prescribed more frequently to Moluccans, as were cardiovascular agents in general (1.27; 0.94–1.71).

**Conclusion:**

The experience of Moluccans in the Netherlands suggests that, in the long run, cardiovascular risk and related health care use of ethnic minority groups may converge towards that of the majority population.

## Introduction

Several studies have shown ethnic differences in mortality of cardiovascular disease.[1–6] In case of a higher incidence and prevalence of cardiovascular disease, special screening and counselling programmes for high-risk ethnic populations may be required. Furthermore, if higher mortality rates result, albeit in part, from unequal access to high-quality healthcare services, then accessibility of quality of health care may need to be improved for these groups. [7, 8]

In the Netherlands, the prevalence of cardiovascular disease is higher among immigrants of Turkish, South Asian and African descent compared to natives [5, 9, 10], whereas Moroccan immigrants showed lower rates of hypertension and cardiovascular disease prevalence, and cardiovascular mortality.[11] Immigrants of African descent in particular have an earlier onset and poorer progression of cardiovascular disease.[5, 9] Furthermore, among Turkish, Surinamese and Antilleans, mortality due to hypertension and cerebrovascular accidents was found to be higher.[12]

The higher prevalence, poorer progression and higher mortality of cardiovascular disease among these ethnic groups raise questions about the accessibility of the Dutch healthcare system. Studies performed in the general population showed different patterns of healthcare use among ethnic minorities. The use of general practitioner (GP) services was relatively high among Surinamese, Turkish and Moroccans.[13–15] In contrast, visits to medical specialist care were less frequent among Turkish and Moroccans compared to native Dutch.[15] However, Surinamese used medical specialist care to the same extent as native Dutch.[15] No previous study focussed on ethnic differences in the utilisation of healthcare services among cardiovascular disease patients.

So far, Moluccan-Dutch residents have been largely neglected in this field of research, even though they have already lived in the Netherlands for over 60 years and thus constitute one of the older non-western ethnic minority groups in Europe. In spring 1951, about 12,500 Moluccan soldiers and their families were forced to move from the Moluccan isles of Indonesia to the Netherlands. [16, 17] Currently, about 50,000 descendants of this cohort live in the Netherlands, most of whom belong to the second and third generation.[16] These people are of particular interest, as it might be expected that 60 years of residence in the Netherlands have removed possible barriers in access to health care, such as low language skills and lack of familiarity with the local health care system.[18]

One health survey among elderly suggested an equal prevalence of cardiovascular disease and equal use of medical specialist care among Moluccans as compared to other Dutch elderly.[1] However, a recent study found the prevalence of hypertension to be higher among Moluccans compared to the native Dutch population.[16] Moreover, a poll held among GPs in the Dutch province of Noord-Brabant suggested cardiovascular disease prevalence to be higher among Moluccan residents.[19] A higher prevalence would be consistent with international variations in cardiovascular risk. International studies suggest that South- and East Asians generally have a higher risk of hypertension, stroke and myocardial infarction.[20–24]

Thus, it remains uncertain whether, after more than 60 years of residence, the Moluccan-Dutch cardiovascular disease profile is comparable to that of native Dutch. Our study therefore aimed to determine the clinical prevalence of cardiovascular disease and hypertension among Moluccans. In addition, we aimed to determine the frequency of visits to the medical specialist and GP and the prescription of cardiovascular agents among Moluccans compared to native Dutch.

We suspected that the cardiovascular health and related health care use of Moluccan residents may still reflect the inequalities that have been observed among groups that immigrated more recently. Therefore, we assumed that the clinical prevalence of cardiovascular disease would be higher among Moluccans compared to the native Dutch. Furthermore, we expected Moluccans with cardiovascular disease, as compared to other Dutch patients, to visit the GP equally often, but to visit medical specialists less often. In accordance with the expected higher clinical prevalence of cardiovascular disease, we expected the cardiovascular agent prescription to be higher among Moluccan patients.

## Methods

### Data collection

We conducted a cross-sectional study using the Achmea Health database with information on health insurance declarations in the period of 1 January 2009 to 31 December 2010. Achmea is the largest health care insurer in the Netherlands, covering approximately 35 per cent of the total Dutch population. As all inhabitants of the Netherlands are obliged to have health insurance, all socioeconomic and ethnic minority groups are represented in the Achmea Health database.

Utilisation of the data was assessed by the local Medical Research Ethics Committee **(**MREC) of the Academic Medical Centre (AMC), Amsterdam. According to the MREC, no approval was required considering the Dutch law on medical research (reference number W13-031# 13.17.0045). All clinical patient records were anonymized and de-identified prior to analysis.

A name list containing Moluccan surnames was used to select the Moluccan population. This list was composed in 1951 when Moluccan families immigrated to the Netherlands by boat. During selection of Moluccans from the Achmea Health database, name variations were taken into account. For privacy reasons, we only used the first four characters of the surnames. A check showed that virtually all Moluccan surnames could be distinguished from Dutch-language surnames on the basis of these four characters.

Moluccans were assessed eligible when they (a) were at least one full year insured at Achmea health insurances and (b) could be identified on the basis of the first four characters of their surnames. Using these criteria, we identified 5,532 Moluccans. These were compared to 55,320 Dutch natives who were randomly extracted from the database, but matched by age and gender. We have composed a control group sized ten times larger in order to have sufficient statistical power.

The acquired database contained the following variables per study subject: gender, age, ethnicity, urbanization, socio-economic status (SES), the number of GP and medical specialist consultations, and the number of days insured. Furthermore, each declared medical procedure was registered in the database by a specific Diagnosis Treatment Combination code (DTC code). Each prescribed (cardiovascular) agent was registered by a specific Anatomical Therapeutic Chemical classification code (ATC code). The level of urbanization was assessed per municipality by Statistics Netherlands on the basis of the number of inhabitants, the population density and the composition of the working population. Municipalities were classified into five categories ranging from ‘rural’ to ‘most urbanized’. SES was determined on the basis of socioeconomic characteristics of the 4-digit postal code areas where patients lived. The area-level SES scores were composed by the Netherlands Institute for Social Research and are widely used in Dutch research.

### Data retrieval

Our primary outcome was the clinical (hospital-based) prevalence of cardiovascular disease (defined as ischemic heart disease, cerebrovascular accidents and cardiac failure) and hypertension (as the major risk factor for cardiovascular disease). For this, we used DTC codes for each disease group and for all cardiovascular diseases together (S1–S5 Tables). On the basis of all DTC codes for each disease the clinical prevalence was determined. We also estimated the prevalence of cardiovascular disease by using both DTC and ATC codes. We therefore composed a list of all ATC codes for agents related to cardiovascular diseases, excluding drugs that are mainly used in the treatment of other diseases (S6 Table).

As secondary outcomes, the numbers of GP and medical specialist consultations were studied for people with hypertension, ischemic heart disease, cerebrovascular accidents and cardiac failure by summing all registered consultations. We only included GP consultations costing more than five euros, thereby excluding all telephone consultations. Neurologist consultations were studied for patients with cerebrovascular accidents and cardiologist consultations for patients with the other three diseases.

An additional secondary outcome was the amount of prescribed agents among Moluccans and native Dutch with clinical cardiovascular disease. We studied prescription of ten agent groups which are predominantly prescribed for cardiovascular disease, as defined by the American Heart Association: ACE inhibitors, angiotensin II inhibitors, anticoagulants, antiplatelet agents, beta blockers, calcium channel antagonists, digitalis preparations, diuretics, statins and vasodilators. [25] The variables for the agent groups were composed using ATC codes (S7 Table).

### Data analysis

We used IBM SPSS Statistics 21.0 to perform analyses. To study the clinical prevalence of cardiovascular disease and hypertension, frequency analyses were performed. Next, logistic regression analyses, controlled for gender, age, urbanization and SES, were conducted to compare Moluccans to native Dutch.

Secondly, we used frequency analyses to determine the mean and standard deviation of the number of GP and medical specialist consultations. To achieve an approximately normal distribution of residuals, we applied logarithmic transformation to the number of consultations. We conducted linear regression analyses controlling for gender, age, urbanization and SES.

Finally, the amount of prescribed agents was analysed using frequency analyses. We conducted logistic regression analyses, controlled for gender, age, urbanization and SES, to compare Moluccans to native Dutch. A p-value ≤0.05 was considered statistically significant.

## Results

Table 1 shows the characteristics of our study populations. The Moluccan population showed a slightly higher degree of urbanization compared to their native Dutch counterparts and lived in similar SES areas.

**Table 1:** Characteristics of the study populations.

Variable	Moluccans	Dutch
	*n* = 5,394	*n* = 52,880
**Women (%)**	50.7	50.8
**Age distribution (%)**		
< 26	30.9	30.9
26–50	35.7	36.6
51–75	29.5	28.8
> 75	3.9	3.7
**Urbanization level (%)**		
Rural	19.2	23.4
Moderately urbanized	15.4	17.6
Highly urbanized	26.9	28.6
Most urbanized	38.5	30.4
Mean SES^a^ (SD^b^)	5.54 (2.88)	5.48 (2.87)

^a^ SES, socio-economic status

^b^ SD, standard deviation.

Table 2 shows the adjusted clinical prevalence’s of different cardiovascular diseases and hypertension. The clinical prevalence of hypertension was higher among Moluccan women compared to Dutch women (1.5% vs. 1.0%; OR = 1.45, 95% CI [1.03, 2.04]). No differences in clinical prevalence of hypertension were found among Moluccan men and among the total Moluccan population. The clinical prevalence of ischemic heart disease was higher in the group of all Moluccans (2.0% vs. 1.6%; OR = 1.26, 95% CI [1.03, 1.56]). The clinical prevalence’s of cerebrovascular accidents and cardiac failure tended to be lower among Moluccan men and among the total Moluccan population, but these differences were not statistically significant. The clinical prevalence of all cardiovascular diseases together tended to be lower among Moluccans (7.2% vs. 8.0%; OR = 0.90, 95% CI [0.80, 1.00]). However, no difference was observed when the prevalence of cardiovascular disease was measured by both DTC and ATC codes.

**Table 2:** Clinical prevalence’s of cardiovascular disease and hypertension: Moluccans compared to Dutch.

Disease	Moluccans (%)	Dutch (%)	OR^a^	(95% CI^b^)	p-value
**Men**					
Hypertension	0.7	1.0	0.73	(0.45, 1.19)	0.21
Ischemic heart disease	2.3	1.8	1.29	(0.98, 1.71)	0.07
Cerebrovascular accidents	0.6	0.9	0.65	(0.38, 1.11)	0.11
Cardiac failure	0.5	0.8	0.73	(0.42, 1.26)	0.25
All cardiovascular diseases	7.0	7.9	0.88	(0.75, 1.04)	0.14
Idem, ATC^c^ codes included	17.0	17.6	0.98	(0.87, 1.11)	0.80
**Women**					
Hypertension	1.5	1.0	1.45	(1.03, 2.04)	0.03
Ischemic heart disease	1.7	1.3	1.24	(0.90, 1.70)	0.19
Cerebrovascular accidents	0.7	0.8	0.93	(0.58, 1.47)	0.74
Cardiac failure	0.3	0.6	0.59	(0.30, 1.17)	0.13
All cardiovascular diseases	7.3	8.1	0.91	(0.78, 1.06)	0.24
Idem, ATC codes included	22.5	21.9	1.07	(0.96, 1.19)	0.22
**Total population**					
Hypertension	1.1	1.0	1.11	(0.84, 1.47)	0.45
Ischemic heart disease	2.0	1.6	1.26	(1.03, 1.56)	0.03
Cerebrovascular accidents	0.6	0.8	0.79	(0.56, 1.11)	0.18
Cardiac failure	0.4	0.7	0.67	(0.44, 1.03)	0.07
All cardiovascular diseases	7.2	8.0	0.90	(0.80, 1.00)	0.06
Idem, ATC codes included	19.8	19.8	1.03	(0.95, 1.12)	0.44

^a^ OR, odds ratio, estimated by logistic regression, controlled for gender, age, urbanization and SES

^b^ CI, confidence interval

^c^ ATC, anatomical therapeutic classification

OR with a p-value ≤ 0.05 is presented in bold.

The mean frequencies of GP visits are shown in Table 3. Moluccans with clinical hypertension visited the GP less often than native Dutch with clinical hypertension (RR = 0.59, 95% CI [0.48, 0.72]). This trend of lower GP visits is also seen for the total Moluccan population (RR = 0.81, 95%CI [0.79,0.83]). Moluccans with clinical ischemic heart disease were more likely to visit the GP than the Dutch (RR = 1.16, 95%CI [0.99,1.34]). No differences were found between Moluccans with cardiac failure and cerebrovascular accidents and their native Dutch counterparts. Moluccans with any clinical cardiovascular disease visited the GP equally often compared to their Dutch counterparts (RR = 0.94, 95% CI [0.87, 1.02]).

**Table 3:** Mean number of GP visits among Moluccans compared to native Dutch.

Subjects with	Number of GP^a^ visits				Difference		
	Moluccans		Dutch		Moluccans versus Dutch		
	Mean	(SD^b^)	Mean	(SD)	RR^c^	(95% CI^d^)	p-value
Hypertension	15.05	(11.23)	22.12	(14.75)	**0.59**	**(0.48, 0.72)**	**0.00**
Ischemic heart disease	26.10	(13.95)	25.56	(17.59)	**1.16**	**(0.99, 1.34)**	**0.05**
Cardiac failure	23.35	(16.88)	29.60	(19.79)	0.73	(0.53, 1.01)	0.06
Cerebrovascular accidents	22.74	(18.83)	26.29	(19.17)	0.80	(0.61, 1.04)	0.09
Any cardiovascular disease	20.61	(15.14)	22.06	(16.99)	0.94	(0.87, 1.02)	0.17
All subjects	9.79	(10.61)	11.32	(12.30)	**0.81**	**(0.79, 0.83)**	**0.00**

^a^ GP, general practitioner

^b^ SD, standard deviation

^c^ RR, relative ratio, estimated by linear regression, controlled for gender, age, urbanization and SES

^d^ CI, confidence interval

RR with a p-value ≤ 0.05 is presented in bold.

The mean frequencies of cardiologist and neurologist visits are shown in Table 4. No evidence was found for substantial differences in cardiologist visits between Moluccans and Dutch with hypertension, ischemic heart disease or cardiac failure. No differences were found either when comparing the total Moluccan population with their native Dutch counterparts (RR = 1.00, 95% CI [0.98, 1.00]). Moluccans with cerebrovascular accidents and the group of all Moluccans were about equally as likely to have neurologist consultations compared to the Dutch (respectively RR = 0.95, 95% CI [0.82, 1.10]; RR = 0.99, 95% CI [0.98, 0.99]).

**Table 4:** Mean number of medical specialist visits among Moluccans compared to Dutch.

Type of visits	Number of visits				Difference		
	Moluccans		Dutch		Moluccans versus Dutch		
	Mean	(SD^a^)	Mean	(SD)	RR^b^	(95% CI^c^)	p-value
**Cardiologist visits**							
Hypertension	1.32	(1.77)	1.63	(1.82)	0.87	(0.73, 1.32)	0.10
Ischemic heart disease	3.35	(2.98)	3.42	(2.63)	0.98	(0.88, 1.08)	0.66
Cardiac failure	3.09	(2.54)	3.72	(3.03)	0.84	(0.68, 1.05)	0.13
All subjects	0.15	(0.75)	0.16	(0.74)	1.00	(0.98, 1.00)	0.41
**Neurologist visits**							
Cerebrovascular accidents	1.54	(0.92)	1.77	(1.30)	0.95	(0.82, 1.10)	0.47
All subjects	0.09	(0.42)	0.11	(0.47)	**0.99**	**(0.98, 0.99)**	**0.04**

^a^ SD, standard deviation

^b^ RR, relative ratio, estimated by linear regression, controlled for gender, age, urbanization and SES

^c^ CI, confidence interval

RR with a p-value ≤ 0.05 is presented in bold.

Table 5 shows the percentages of patients with cardiovascular disease using a prescribed cardiovascular agent belonging to one of the ten agent categories. Angiotensin II inhibitors were prescribed to Moluccans more frequently than to Dutch (OR = 1.42; 95% CI [1.09–1.84]), as were antiplatelet agents (OR = 1.27; 95% CI [1.01–1.59]) and statins (OR = 1.27; 95% CI [1.00–1.60]). No significant differences were observed for other cardiovascular agent groups. Overall, cardiovascular agents tended to be more commonly prescribed to Moluccans than to native Dutch (OR = 1.27; 95% CI [0.94–1.71]).

**Table 5:** Percentage of Moluccan cardiovascular patients receiving cardiovascular agents compared to Dutch patients.

Agent category	Patients receiving this agent				
	Moluccans	Dutch	Difference		
	(*n* = 388)	(*n* = 4,219)	Moluccans versus Dutch		
	(%)	(%)	OR^a^	(95% CI^b^)	p-value
ACE inhibitors	29.1	27.5	1.13	(0.89, 1.43)	0.31
Angiotensin II inhibitors	22.2	17.6	**1.42**	**(1.09, 1.84)**	**0.01**
Anticoagulants	1.3	3.2	0.42	(0.17, 1.03)	0.06
Antiplatelet agents	45.4	41.0	**1.27**	**(1.01, 1.59)**	**0.04**
Beta blockers	46.1	45.7	1.05	(0.84, 1.31)	0.67
Calcium channel antagonists	26.8	23.5	1.24	(0.97, 1.58)	0.08
Digitalis preparations	3.6	3.4	1.13	(0.64, 2.00)	0.67
Diuretics	29.1	27.8	1.10	(0.87, 1.41)	0.43
Statins	48.5	44.5	**1.27**	**(1.00, 1.60)**	**0.04**
Vasodilators	18.6	16.6	1.18	(0.90, 1.56)	0.24
All categories together	76.8	75.2	1.27	(0.94, 1.71)	0.13

^a^ OR, odds ratio, estimated by logistic regression, controlled for gender, age, urbanization and SES

^b^ CI, confidence interval

OR with a p-value ≤ 0.05 is presented in bold.

## Discussion

Our study investigated differences between Moluccans and native Dutch in the clinical prevalence’s of cardiovascular disease and hypertension, and the use of related health care services. The relative importance of different cardiovascular diseases was found to vary between Moluccans and native Dutch. Among Moluccans, we found significantly higher clinical prevalence of hypertension among Moluccan women and higher prevalence of ischemic heart disease for the group of all Moluccans. We also found a significantly lower amount of GP visits among Moluccans with hypertension and among the group of all Moluccans. No substantial differences were found regarding cardiologist and neurologist consultations. Finally, prescription of angiotensin II inhibitors, antiplatelet agents and statins was more common among Moluccans.

A limitation of our study is that the prevalence’s of cardiovascular disease and hypertension were determined using DTC codes based on hospital care. If Moluccans are less likely than native Dutch to visit hospitals in case of disease, like other ethnic minorities as Turkish and Moroccans [15], the prevalence of disease would be underestimated to a greater extent among Moluccans than among native Dutch. We aimed to reduce this effect by also determining the prevalence of total cardiovascular disease using ATC as well as DTC codes. ATC-based measures may overestimate prevalence rates because some of the agents prescribed for cardiovascular disease are also prescribed for other diseases. However, this would apply to both Moluccan and native Dutch.

Unfortunately, the number of Moluccans with specific cardiovascular diseases was, at times, too small to estimate Moluccan-Dutch differences with precision. Therefore we also estimated the differences for the total of cardiovascular diseases in both groups.

The main results do not support our initial hypothesis of a generally higher clinical prevalence of cardiovascular disease among Moluccans compared to the native Dutch. The tendency of a lower prevalence of cardiovascular disease may be related to a lower prevalence of some key risk factors. A recent health survey showed no differences regarding BMI and physical activity level between Moluccans and native Dutch. However, a lower prevalence of heavy smoking and excessive alcohol drinking was found among Moluccans. [16]

The results do not support our hypothesis regarding a similar frequency of GP visits and fewer specialist consultations among Moluccans. We instead found for Moluccans to visit the GP less frequently, a finding that corresponds to a recent study of Verhagen et al., which found systematically lower health care usage among Moluccans compared to the native Dutch.[26] Regarding specialists consultations, cardiologist visits were only slightly less common among Moluccans, and neurologist consultations were equally common among Moluccans compared to the native Dutch. This pattern might be specific to the care for cardiovascular patients, where the importance of adhering to clinical guidelines might overrule potential inequalities in the use of health care.[27]

We expected more prescription of cardiovascular agents to Moluccans compared to native Dutch. Our study indeed showed higher prescription of medication from some of the cardiovascular agent groups. More frequent prescription of angiotensin II inhibitors may relate to hypertension, which is in agreement with a recent Dutch study that showed a higher prevalence of self-reported hypertension among Moluccans. [16] The more frequent prescription of antiplatelet agents and statins among Moluccans compared to native Dutch may relate to the higher clinical prevalence of ischemic heart diseases among Moluccans.

Because of the limited amount of studies specifically looking into the cardiovascular health and health care use of ethnic groups in the Netherlands, it is difficult to assess whether our results are consistent with those for other ethnic minorities. Studies from other countries on long-residing ethnic minorities have found to some extent similar results. A US study on first generation Chinese immigrants found lower cardiovascular diseases, possibly due to maintenance of a healthier lifestyle after two decades of residence in the US. [28] A study from Israel found total convergence towards the national level of hypertension prevalence among African immigrants after 20 years of residence in Israel. [29]

We observed no substantial and consistent differences between Moluccans and native Dutch in the frequency of visits to medical specialists. Differences in agent prescription were consistent with differences in the relative importance of different cardiovascular diseases. Our results may suggest equal specialist health care access among Moluccans, as they are familiar with the host language and health care system. This pattern of health care use may indicate proper referrals to medical specialists of Moluccans in case of cardiovascular disease.

Several studies documented large variations in cardiovascular risk in relationship to ethnic minority status. [28–32] Yet, the experience of Moluccans in the Netherlands suggests that, in the long run, cardiovascular risk may converge towards levels of the majority population. Their experience also illustrates that, under equitable health care systems as in the Netherlands, the use of health care services may in the long run become similar to that of the host population. Nonetheless, convergence cannot be taken for granted. Therefore, ongoing attention is needed to cardiovascular health and related health care use of ethnic minority groups, particularly of those who arrived more recently.

## Supporting Information

S1 Table DTC codes stroke. (DOC)

S2 Table DTC codes hypertension. (DOC)

S3 Table DTC codes ischemic heart disease. (DOC)

S4 Table DTC codes cardiac failure. (DOC)

S5 Table DTC codes all other cardiovascular diseases. (DOC)

S6 Table Included ATC codes per medication category. (DOC)

S7 Table Included medication categories for medication prescription analysis. (DOC)
