# Supplementary material for: Cardiovascular Health and Related Health Care Use of Moluccan-Dutch Immigrants
Source: PLoS One. 2015 Sep 22;10(9):e0138644. doi: 10.1371/journal.pone.0138644 (PMC4578883; doi:10.1371/journal.pone.0138644)
Supplement: S1 Table — (DOC) [file pone.0138644.s001.doc]

**Supporting Information Caption**

S1 table: DTC codes stroke

| **Diagnosis code** | **Specialism code** | **Description** |
| --- | --- | --- |
| 1101 | 0330 | Subarachnoid haemorrhage |
| 1102 | 0330 | Intracerebral haemorrhage |
| 1103 | 0330 | Intracranial haemorrhage (sub-/epid.) |
| 1111 | 0330 | Bloodless stroke |
| 1112 | 0330 | TIA (incl. amaurosis fugax) |
| 1121 | 0330 | Remainder (brain injury) |
| 9927 | 0330 | Hypoth. diagnosis TIA |
| 0121 | 0313 | Stroke/ TIA |
| 0213 | 0327 | Stroke, incl. infections/ tumours |
| 0215 | 0327 | Sequelae stroke |
| 0263 | 0335 | Stroke/ TIA |
| 0313 | 0327 | Stroke |
| 1199 | 0330 | Stroke (other) |
| 3508 | 0316 | Intracranial haemorrhage |
| 3501 | 0316 | Cerebral infarction |
| 2201 | 0308 | Spine, epi- or subdural haemorrhage |
| 3501 | 0316 | cerebral infarction |
| 3508 | 0316 | Intracranial haemorrhage |
| 1230 | 0308 | Intracerebral supratentorial hematoma |
| 1235 | 0308 | Intracerebral infratentorial hematoma |
| 1240 | 0308 | Decompression stroke |
| 1315 | 0308 | Epidural/subacute subdural hematoma |
| 1321 | 0308 | Chronic subdur hematoma/hygroma |
| 9925 | 0330 | Hypoth. subarachnoidal hemorrhage |
