# Supplementary material for: Cardiovascular Health and Related Health Care Use of Moluccan-Dutch Immigrants
Source: PLoS One. 2015 Sep 22;10(9):e0138644. doi: 10.1371/journal.pone.0138644 (PMC4578883; doi:10.1371/journal.pone.0138644)
Supplement: S2 Table — (DOC) [file pone.0138644.s002.doc]

**Supporting Information Caption**

**S2 table: DTC codes hypertension**

| **Diagnosis code** | **Specialism code** | **Description** |
| --- | --- | --- |
| 0902 | 0320 | Hypertension |
| 0901 | 0318 | Hypertension |
| 4003 | 0316 | Hypertension |
| 0311 | 0313 | Hypertension |
