# Supplementary material for: Cardiovascular Health and Related Health Care Use of Moluccan-Dutch Immigrants
Source: PLoS One. 2015 Sep 22;10(9):e0138644. doi: 10.1371/journal.pone.0138644 (PMC4578883; doi:10.1371/journal.pone.0138644)
Supplement: S3 Table — (DOC) [file pone.0138644.s003.doc]

**Supporting Information Caption**

**S3 table: DTC codes ischemic heart disease**

| **Diagnosis code** | **Specialism code** | **Description** |
| --- | --- | --- |
| 0101 | 0313 | Symp. isch. heart disease, |
| 0102 | 0313 | Instable AP, myocardial infarction |
| 0202 | 0320 | Angina pectoris stable |
| 0204 | 0320 | ST elevated myocardial infarction |
| 0205 | 0320 | Non-ST elevated myocardial infarction |
| 0405 | 0318 | Ischemia? |
| 0911 | 0320 | Heart team meeting |
| 2110 | 0328 | Heart team meeting without surgery |
| 0101 | 0313 | Symp. isch. heart disease, not DBC-102 |
| 0202 | 0320 | Angina pectoris, stable |
| 0203 | 0320 | Angina pectoris, instable |
