# Supplementary material for: Cardiovascular Health and Related Health Care Use of Moluccan-Dutch Immigrants
Source: PLoS One. 2015 Sep 22;10(9):e0138644. doi: 10.1371/journal.pone.0138644 (PMC4578883; doi:10.1371/journal.pone.0138644)
Supplement: S4 Table — (DOC) [file pone.0138644.s004.doc]

**Supporting Information Caption**

**S4 table: DTC codes cardiac failure**

| **Diagnosis code** | **Specialism code** | **Description** |
| --- | --- | --- |
| 0107 | 0313 | Congestive heart failure |
| 0262 | 0335 | Congestive heart failure |
| 0302 | 0320 | Chronic heart failure |
| 3406 | 0316 | Congestive heart failure |
| 0301 | 0320 | Acute congestive heart failure |
