# Supplementary material for: Cardiovascular Health and Related Health Care Use of Moluccan-Dutch Immigrants
Source: PLoS One. 2015 Sep 22;10(9):e0138644. doi: 10.1371/journal.pone.0138644 (PMC4578883; doi:10.1371/journal.pone.0138644)
Supplement: S5 Table — (DOC) [file pone.0138644.s005.doc]

**Supporting Information Caption**

**S5 table: DTC codes all other cardiovascular diseases**

| **Diagnosis code** | **Specialism code** | **Description** |
| --- | --- | --- |
| 0106 | 0313 | AF, rhythm and conduction dis. |
| 0124 | 0313 | Atheroscl. extr./ peripheral art. dis. |
| 0129 | 0313 | Aneurysm and other art./vessel |
| 0261 | 0335 | Cardiovascular disease |
| 0311 | 0362 | Heart |
| 0401 | 0320 | Atrial fibrillation/ flutter |
| 0403 | 0303 | Aneurysm thor. aorta, rupture |
| 0403 | 0320 | Ventricular arrhythmias |
| 0404 | 0320 | Impulse and conduction dis. |
| 0405 | 0303 | Aneurysm iliac aorta |
| 0406 | 0303 | Aneurysm abdominal aorta, rupture |
| 0409 | 0320 | Other arrhythmias |
| 0439 | 0303 | Other peripheral arterial disease |
| 0502 | 0320 | Congen. cardiovascular malformations |
| 0510 | 0327 | Cardiovascular disease |
| 0609 | 0320 | Other cardiovascular disease |
| 0613 | 0327 | Heart disorders |
| 0614 | 0327 | Blood vessels |
| 0821 | 0320 | Heart revalidation |
| 0907 | 0318 | Cardiology |
| 0911 | 0320 | Heart team meeting |
| 1102 | 0322 | Chest pain |
| 1201 | 0308 | Conservative treatment +/- coiling |
| 1703 | 0316 | Cardiomyopathy |
| 2110 | 0328 | Heart team meeting without surgery |
| 2120 | 0328 | Heart consult without surgery |
| 2210 | 0328 | Drainage pericardium |
| 2311 | 0316 | Heart and blood vessels |
| 2312 | 0316 | Heart and blood vessels, severe |
| 2315 | 0328 | Heart trauma (LV rupture) |
| 3410 | 0316 | Arrhythmias |
| 3499 | 0316 | Other cardiovascular diseases |
| 3523 | 0316 | Syncope/collaps/non-epilept. Insult |
| 2210 | 0308 | Vasc. dis. spine/spinal cord: cons. tr. |
| 2220 | 0328 | Pacemaker implantation |
| 2230 | 0328 | AICD implantation |
| 2240 | 0328 | Pacemaker lead extractive |
| 2250 | 0328 | Restenometry |
| 2260 | 0328 | Insertion IABP |
| 2310 | 0328 | Construction Blalock shunt |
| 2320 | 0328 | CABG, ven. graft, max. 1 art. graft |
| 2325 | 0328 | Aortic valve replacement (AVR) |
| 2335 | 0328 | Mitral valve replacement (MVR) |
| 2340 | 0328 | Pulmonary valve plasty/replacement |
| 2345 | 0328 | ASD closure |
| 2350 | 0328 | VSD closure |
| 2360 | 0328 | Left ventricle plastic |
| 2370 | 0328 | Coarctatio aortae(-ECC) restoration |
| 2380 | 0328 | Ductus Botalli closure |
| 2390 | 0328 | Impl. bipolar pacemaker electrode |
| 2400 | 0328 | CABG (?2 art. grafts) |
| 2405 | 0328 | Mitral valve plasty (MPL) |
| 2410 | 0328 | Tricuspid valve plasty/replacement |
| 2415 | 0328 | CABG (1 art.) + MVR |
| 2420 | 0328 | AVR + MVR |
| 2425 | 0328 | CABG (1 art.) + AVR |
| 2430 | 0328 | LVAD connection |
| 2435 | 0328 | Tetralogy of Fallot |
| 2440 | 0328 | AVSD complete |
| 2445 | 0328 | Pericardiectomy |
| 2460 | 0328 | Reanimation with ECC +/- LVAD |
| 2470 | 0328 | Left ventricle plasty + CABG |
| 2475 | 0328 | Left ventricle plasty + AVR/MVR |
| 2510 | 0328 | Aortic trunk replacement |
| 2515 | 0328 | Arterial switch operation |
| 2520 | 0328 | Ventricle septal rupture |
| 2525 | 0328 | Maze procedure |
| 2540 | 0328 | TAPVC (abn. pulmonary vein con.) |
| 2550 | 0328 | CABG + MPL +/- TPL |
| 2555 | 0328 | CABG (2 art.) + MVR |
| 2565 | 0328 | AVR + MVR + TPL |
| 2610 | 0328 | Ascending aorta replacement |
| 2615 | 0328 | Aortic arch surgery |
| 2630 | 0328 | VT + CABG |
| 2635 | 0328 | Maze + CABG |
| 2640 | 0328 | VSR + CABG |
| 2645 | 0328 | MPL + AVR + CABG |
| 2650 | 0328 | MPL + CABG (2 art) |
| 2655 | 0328 | AVR + CABG + HOCM |
| 2660 | 0328 | Aortic trunk + MVR/MPL |
| 2665 | 0328 | Aortic trunk + CABG |
| 2670 | 0328 | Other complex cong. dis. |
| 2675 | 0328 | Maze + MVP/MPL +/- TPL |
| 2680 | 0328 | AVR + ascending aorta |
| 2695 | 0328 | AVR + MVR +/- TPL |
| 2710 | 0328 | Pulm. thromboendarteriectomy |
| 2720 | 0328 | Aortic dissection +/- CABG |
| 2730 | 0328 | Aortic arch + aortic trunk + ao. asc. |
| 2740 | 0328 | Ao. asc. + CABG |
| 2750 | 0328 | Ao. asc. + valve reconstruction |
| 2760 | 0328 | Ao. asc. + (part.) arch |
| 2765 | 0328 | Ao. desc. replacement |
| 2770 | 0328 | Ao. trunk + CABG + MPL/MVR |
| 2775 | 0328 | Ao. dissection, tr./conservative |
| 2910 | 0328 | Heart transplantation |
| 2930 | 0328 | Heart-lung transplantation |
| 2940 | 0328 | Art. heart implantation |
| 3330 | 0328 | Iliac aneurysm |
| 3402 | 0316 | Cor vitium, cyanotic |
| 3403 | 0316 | Cor vitium, non-cyan., hemod. imp. |
| 3404 | 0316 | Cor vitium, non-cyan., hemod. unimp. |
| 3405 | 0316 | Cor vitium, after cardiac surgery |
| 3499 | 0316 | Other cardiac dis. |
| 7406 | 0316 | Vascular dis. |
| 0103 | 0313 | Pericarditis |
| 0104 | 0313 | Myocarditis |
| 0432 | 0313 | endocarditis / endovasc. infiltration |
| 0701 | 0320 | Pericarditis |
| 0702 | 0320 | Endocarditis |
| 3407 | 0316 | Endocarditis |
| 3408 | 0316 | Myocarditis / cardiomyopathy |
| 1803 | 0322 | Primary pulmonary hypertension |
| 0125 | 0313 | Pulmonary hypertension |
| 0402 | 0320 | Other suprav. arrhythmias |
| 0801 | 0320 | Follow-up after acute cor syndr. |
| 0802 | 0320 | Follow-up after PTCA a/o CABG/abl |
| 0803 | 0320 | Follow-up after PM implantation |
| 0806 | 0320 | Follow-up after surg. heart valve dis. |
| 0807 | 0320 | F-up after surg. con. cardiovascular dis. |
| 0909 | 0320 | Other disorders (cardiovascular) |
| 2311 | 0316 | Heart and blood vessel |
| 2312 | 0316 | Heart and blood vessel, severe |
| 0010 | 0390 | Congenital disease of circulatory tract |
| 0013 | 0389 | Vascular headache |
| 0024 | 0310 | Varices |
| 0101 | 0362 | A. carotis interna flow area |
| 0102 | 0362 | A. carotis externa flow area |
| 0119 | 0313 | Other cardiovascular deformities |
| 0312 | 0362 | Pericardium |
| 0402 | 0303 | Carotis pathology |
| 0408 | 0303 | A. renalis stenosis |
| 0409 | 0303 | Vascular abnormalities abdomen/pelvis |
| 0418 | 0303 | P.A.O.D. 2, claudicatio intermittens |
| 0419 | 0303 | P.A.O.D. 3, rest pain |
| 0420 | 0303 | P.A.O.D. 4, gangrene |
| 0423 | 0303 | Varices lower extremities |
| 0449 | 0303 | Other vascular diagnoses |
| 0501 | 0320 | Cardiac valve deformities |
| 0509 | 0320 | Other structural deformities heart |
| 0601 | 0320 | Arterial deformities / stenosis |
| 0602 | 0320 | Venous deformities |
| 0709 | 0320 | Other cardiovascular inflammations |
| 0804 | 0320 | Follow-up after ICD implantation |
| 0805 | 0320 | Follow-up after heart transplantation |
| 0808 | 0320 | Follow-up after vascular surgery |
| 0810 | 0320 | Follow-up after other cardiac surgery |
| 0822 | 0320 | Perioperative care |
| 0901 | 0320 | Fat metabolism disorders |
| 0912 | 0320 | Screening heart transplantation |
| 1205 | 0308 | Surgery unil. non-complex aneurysm |
| 1210 | 0308 | Surgery complex aneurysma |
| 1701 | 0316 | Open ductus Botalli |
| 1704 | 0316 | arrhythmia |
| 1801 | 0322 | Vascular disease |
| 2101 | 0322 | Primary cardiac disease |
| 2205 | 0308 | Vascular malformation spine |
| 2365 | 0328 | AVSD partial |
| 2375 | 0328 | PM lead extraction with ECC |
| 2450 | 0328 | HOCM (Morrow procedure) |
| 2465 | 0328 | MVR and TPL |
| 2530 | 0328 | VT endocardium resection |
| 2535 | 0328 | Cavo-pulmonary con. partial/total |
| 2560 | 0328 | CABG (2 art.) and AVR |
| 2575 | 0328 | AVR and MPL |
| 2580 | 0328 | AVR and HOCM |
| 2585 | 0328 | CABG and HOCM |
| 2590 | 0328 | MPL and TPL |
| 2620 | 0328 | Norwood surgery |
| 2625 | 0328 | Truncus correction |
| 2780 | 0328 | Thoracic endoprothesis |
| 2785 | 0328 | Maze, CABG or AVR, MPL, TPL |
| 2790 | 0328 | AVR, ascendens, MPL, TPL |
| 2810 | 0328 | Thoraco-abdominal aneurysm |
| 3110 | 0328 | Arteriovenous fistula |
| 3120 | 0328 | False inguinal aneurysm |
| 3210 | 0328 | Carotis endarteriectomy (CEA) |
| 3220 | 0328 | Other arcuate artery surgery |
| 3310 | 0328 | CEA bilateral |
| 3320 | 0328 | AAA |
| 3340 | 0328 | Endoprothesis |
| 3401 | 0316 | Cardiac murmur, benign |
