# Supplementary material for: Cardiovascular Health and Related Health Care Use of Moluccan-Dutch Immigrants
Source: PLoS One. 2015 Sep 22;10(9):e0138644. doi: 10.1371/journal.pone.0138644 (PMC4578883; doi:10.1371/journal.pone.0138644)
Supplement: S6 Table — (DOC) [file pone.0138644.s006.doc]

**Supporting Information Caption**

| **Medication category** | **Included subgroups** |
| --- | --- |
| C01 cardiacs | C01A, C01B, C01C, C01D, C01E |
| C02 antihypertensives | C02A, C02B, C02C, C02D, C02K, C02L, C02N |
| C03 diuretics | C03A, C03B, C03C, C03D, C03E |
| C04 peripheral vasodilators | C04A |
| C05 vasoprotectives | C05B, C05C |
| C07 beta blockers | C07A, C07B, C07C, C07D, C07E, CO7F |
| C08 calcium channel antagonists | C08C, C08D, C08E, C08G |
| C09 RAAS inhibitors | C09A (except C09AA01, C09AA05 and C09AA07), C09B, C09C, C09D, C09X |
| C10 antilipemics | C10BX |

**S6 table: Included ATC codes per medication category**
