# Supplementary material for: Cardiovascular Health and Related Health Care Use of Moluccan-Dutch Immigrants
Source: PLoS One. 2015 Sep 22;10(9):e0138644. doi: 10.1371/journal.pone.0138644 (PMC4578883; doi:10.1371/journal.pone.0138644)
Supplement: S7 Table — (DOC) [file pone.0138644.s007.doc]

**Supporting Information Caption**

| **Medication category** | **Included subgroups** |
| --- | --- |
| ACE inhibitors | C09A (except C09AA01, C09AA05 and C09AA07), C09B |
| Angiotensin II inhibitors | C09C, C09D |
| Anticoagulants | B01AB |
| Antiplatelet agents | B01AC |
| Beta blockers | C07A, C07B, C07C, C07D, C07E, CO7F |
| Calcium channel antagonists | C08C, C08D, C08E, C08G |
| Digitalis preparations | C01AA |
| Diuretics | C03A, C03B, C03D, C03E |
| Vasodilators | C01D, C02D (except C02DA) |
| Statins | C10A (except C10AC), C10B |

**S7 table: Included medication categories for medication prescription analysis**
